# Risk factors of early childhood caries among children in Beijing: a case-control study

**DOI:** 10.1186/s12903-016-0289-6

**Published:** 2016-09-17

**Authors:** Cancan Fan, Wenhui Wang, Tao Xu, Shuguo Zheng

**Affiliations:** Department of Preventive Dentistry, Peking University School and Hospital of Stomatology, National Engineering Laboratory for Digital and Material Technology of Stomatology, Beijing Key Laboratory of Digital Stomatology, 22 Zhongguancun Avenue South, Haidian District Beijing, 100081 People’s Republic of China

**Keywords:** Early childhood caries, Mutans streptococci, Risk factors

## Abstract

**Background:**

The prevalence of early childhood caries (ECC) among children in Beijing, China, has been increasing continuously though slowly. However, there is limited information about ECC in Beijing. The aim of this study was to identify risk factors of dental caries among preschool children in Beijing.

**Methods:**

For this case-control study, using a convenience sampling method, 787 children aged 3 and 4 years old were recruited; 386 children with caries constituted the early childhood caries (ECC) group and 401children without caries formed the caries-free (CF) group. Dental caries was diagnosed at the tooth surface level by two calibrated examiners according to the WHO 1997 criteria. A structured questionnaire was filled in by the children’s main guardians. Mutans streptococci in non stimulated saliva and plaque were measured with the Dentocult SM Strip. Negative binomial regression was used for multivariate analysis.

**Results:**

Analysis of the data showed that level of mutans streptococci in dental plaque and history of dental visit were significantly correlated with the prevalence of caries and the mean dmfs score.

**Conclusions:**

High level of plaque mutans streptococci is a risk factor for ECC in preschool children in Beijing. And longitudinal studies are needed to identify the causal relationships between the levels of mutans streptococci in dental plaque and caries development.

## Background

Although preventive strategies have been implemented for decades, dental caries remains a major problem in both developed and developing countries [1, 2]. The prevalence of early childhood caries (ECC) is especially high in developing countries [3]. Among the possible causes of ECC that have been identified are poor oral hygiene (i.e. visible dental plaque), inadequate tooth brushing, dietary habits (i.e. consumption of sugary snacks and frequency of between-meal snacks), and the presence of specific pathogens [4, 5]. The risk factors appear to be different in children from different backgrounds [6, 7]. Among these factors, however, mutans streptococci are a common factor that is strongly associated with carious lesions [8, 9]. Saliva was sampled to measure cariogenic bacteria in most studies, but there are only a few studies that sampled both saliva and plaque simultaneously [10, 11].

The prevalence of ECC among children in Beijing has been increasing continuously though slowly. An oral health survey of Beijing residents in 2010 showed that the prevalence of caries among children age groups at 2, 3, 4 and 5 years old were 20, 40, 55 and 66 %, respectively [12]. A 2005 study reported the prevalence of caries in 5-year-old children was 58.6 % [13]. However, there are few studies on the prevalence and risk factors of ECC in children under 5 years of age in Beijing. Therefore, the aim of this study was to investigate the factors associated with ECC in children in Beijing, China.

## Methods

### Research design and participants

The present research is a part of our longitudinal studies of factors related to the incidence and development of ECC in Beijing. This case-control study was conducted in Haidian District of Beijing in 2013. The fluoride concentration in the drinking water is around 0.3–0.4 mg⁄L. There is no any systemic use of fluoride in China. All preschool children in kindergartens in Beijing undergo 1.23 % fluoride foam twice per year. The sample size was estimated according to the prevalence (40 %) of 3-year old children in Beijing reported in 2010 [12]. Therefore, to analyze 17 variables, the total sample size of study children was about 369 using the formula N=Z2 ×(P ×(1-P)) / E2, with a margin of error of 5 % and a confidence level of 95 %.

A convenience sampling method was used: all 842 children aged 3 and 4 years old from six kindergartens near our hospital were invited to participate in this study. Children who had taken antibiotics within the 2 weeks preceding the examination were conducted a few weeks later. The project was approved by the Human Research Ethics Committee of the School of Stomatology, Peking University, China (PKUSSIRB -2012042).

### Oral health questionnaires

Details regarding the children’s demographic status (gender and birth date), nursing feeding habits, sucrose diet habits, oral hygiene practices (brushing frequency, guardians’ help, and type of toothpaste), dental visit history, and the guardians’ educational level were obtained through a structured questionnaire filled in by the main guardians (Table 1). The questionnaire was simplified from the third national oral health survey in China [14]. Written informed consent for participation in this research was obtained from the guardians of each child included in this study.

**Table 1:** Characteristics of participants and results of laboratory tests

Variables	ECC group *N* (%)	CF group *N* (%)	Total	*P*
Age (months)	46.3 ± 5.43	45.7 ± 5.73	787	0.96
Gender				0.09
Male	220 (58.0)	204 (49.6)	424	
Female	166 (42.0)	197 (50.4)	363	
Person filling out the questionnaire				0.08
Father	102 (26.4)	92 (22.9)	194	
Mother	238 (61.7)	267 (66.6)	505	
Grandfather/Grandmother	42 (10.9)	40 (10.0)	82	
Other (relative/nanny)	4 (1.0)	2 (0.5)	6	
Using nursing bottle or not at present				0.01*
Yes	70 (18.2)	102 (25.4)	172	
No	315 (81.8)	299 (74.6)	614	
Contents of nursing bottle				0.78
Water or milk	155 (87.1)	152 (89.4)	307	
Sugared beverages	21 (12.9)	18 (10.6)	39	
Sleeping with nursing bottle				0.45
Always	8 (4.1)	3 (1.5)	11	
Sometimes	24 (12.3)	23 (11.3)	47	
Rarely or never	163 (83.6)	178 (87.2)	341	
Frequency of snack consumption				0.03*
Never or seldom	22 (5.7)	30 (7.5)	52	
Sometimes (<1 Times/Day)	93 (24.3)	94 (23.5)	187	
1–2 Times/Day	166 (43.3)	204 (50.8)	370	
3–4 Times/Day	86 (22.5)	65 (16.2)	151	
≥5 Times/Day	16 (4.2)	8 (2.0)	24	
Rinsing or brushing teeth after snacks				0.71
Always	104 (27.1)	114 (28.4)	218	
Sometimes	173 (45.2)	183 (45.6)	356	
Never or seldom	106 (27.7)	104 (26.0)	210	
Sleeping without brushing after snacks				0.70
Always	22 (5.8)	22 (5.5)	44	
Sometimes	96 (25.1)	94 (23.5)	190	
Never or seldom	264 (69.1)	284 (71.0)	548	
Frequency of tooth brushing				0.008*
Twice daily	185 (48.4)	156 (39.2)	341	
Once daily	161 (42.2)	176 (44.2)	337	
Less than once daily	32 (8.4)	58 (14.6)	90	
Never	4 (1.0)	8 (2.0)	12	
Guardians helping with the brushing				0.23
Everyday	116 (30.4)	97 (24.4)	213	
Sometimes	137 (35.9)	144 (36.2)	281	
No or never brushing	129 (33.7)	157 (39.4)	286	
Toothpaste containing fluoride				0.27
Yes	151 (39.8)	136 (34.7)	287	
No	122 (32.2)	127 (32.4)	249	
Not sure	87 (23.0)	99 (25.3)	186	
Not having used toothpaste	19 (5.0)	30 (7.6)	49	
Dental visit history				<0.001**
Yes	279 (73.2)	121 (30.4)	400	
No	102 (26.8)	277 (69.6)	379	
Education level of person filling out the questionnaire				0.07
Junior high school	6 (1.6)	10 (2.5)	16	
Senior high school	104 (27.3)	87 (21.8)	192	
College and above	271 (71.1)	303 (75.7)	576	
MS score of saliva				<0.001**
0 (CFU <10^4^)	152 (39.4)	306 (76.3)	458	
1 (CFU 10^4^–10^5^)	67 (17.4)	50 (12.5)	117	
2 (CFU 10^5^–10^6^)	124 (32.1)	39 (9.7)	163	
3 (CFU >10^6^)	43 (11.1)	6 (1.5)	49	
MS score of plaque				<0.001**
0 (CFU <10^4^)	80 (20.7)	243 (60.6)	323	
1 (CFU 10^4^–10^5^)	16 (4.2)	19 (4.7)	35	
2 (CFU 10^5^–10^6^)	34 (8.8)	39 (9.7)	73	
3 (CFU >10^6^)	256 (66.3)	100 (25)	356	

*MS* mutans streptococci

*Significant at *P* <0.05; **Significant at *P* <0.001

### Dental examination

Dental examinations were conducted under a portable light by two calibrated examiners using a CPI (community periodontal index) explorer and a disposable mirror, with a cotton swab to dry the teeth. The child, seated in a small chair, was asked to recline on to the knee of the examiner. Dental caries was diagnosed at the tooth surface level according to the WHO 1997 criteria [15], CPI probe was applied with visual examination in diagnosis. Radiography was not used in this study. The inter-examiner kappa value was 0.82 and intra-examiner kappa values for the two examiners were 0.88 and 0.86. Children were reexamined by two examiners 1 week later for the calculation of intra-examiner kappa values.

### Plaque and saliva samples

Dentocult SM Strip (Orion Diagnostica, Espoo, Finland) was used to evaluate the levels of mutans streptococci in both non stimulated saliva and plaque [16, 17]. The non stimulated saliva samples were obtained by pressing the rough surface of the strip against the child’s tongue and turning it over 10 times. The children were not asked to chew paraffin for salivary stimulation. Four specific sites of dental plaque were sampled: the buccal surfaces of teeth 55, 51, and 71, and the lingual surface of tooth 75. Plaque samples were collected by stroking the tooth surface near the gingival margin with a mini-brush; separate brushes were used for each tooth. The brushes with the plaque samples from the four teeth were immediately applied to the four roughened sites on the strip, and the strips were then placed in the culture medium and incubated at 37 °C for 48 h.

The level of mutans streptococci was scored following the standard provided by the manufacturer. The score “0” corresponds to <10^4^ of CFU (colony-forming units)/mL, “1” to 10^4^–10^5^ of CFU/mL, “2” to 10^5^–10^6^ of CFU/mL, and “3” to >10^6^ of CFU/mL. The scores of all strips were recorded by the same investigator, to whom the results of clinical examination were not available. The highest score of the four sites was recorded as the plaque mutans streptococci level of the child.

### Statistical analyses

The research data included the results of the dental examination, the bacterial scores assessed by the Dentocult SM Strip method, and the information from the questionnaire survey. The chi-square test or Fisher’s exact test were used in univariate analyses to assess the differences between the ECC and CF groups. The mean dmfs scores of children with the same level of mutans streptococci infection were calculated and were compared using the t-test and one-way ANOVA using SPSS Statistics for Windows, version 20.0 (IBM Corp. Armonk, NY, USA). Negative binomial regression was used to identify the variables associated with ECC; SAS software, version 9.3 (SAS Institute, Cary, NC, USA) was used for this analysis. Statistical significance was set at *P* <0.05.

## Results

### Demographics

In all, 787 children aged between 26 and 57 months (mean age 46.02 ± 5.59) responded, submitting the filled-in questionnaires and undergoing dental examination and laboratory test. Four hundred and twenty-four (53.9 %) were male. The caries prevalence and mean dmfs score of these children were 49 % and 4.50 ± 7.92. These children were separated into two groups: a caries (ECC) group, comprising 386 children with dmfs ≥1, and a caries-free (CF) group, comprising 401 children without visible diagnosed caries.

### Univariate analyses results

The information obtained through the questionnaire and the scores for mutans streptococci infection in the two groups are presented in Table 1. Six variables, i.e., use of nursing bottle or not at present (*P* = 0.01), frequency of snacks consumption (*P* = 0.03), Frequency of tooth brushing (*P* = 0.008), dental visit history (*P* <0.001), plaque mutans streptococci level (*P* <0.001), and salivary mutans streptococci (*P* <0.001) were associated with dental caries by univariate analyses.

In the ECC group, 60.6 % of children scored ≥1 for salivary mutans streptococci, compared to 23.7 % in the CF group; for plaque mutans streptococci, 79.3 % scored ≥1 in the ECC group vs. 39.4 % in the CF group.

### Negative binomial regression test

Of the six variables that were found to be significantly associated with caries in univariate analysis, only mutans streptococci in plaque and dental visit history retained statistically significant association with dental caries on multivariate analysis (Table 2).

**Table 2:** Results of negative binomial regression for dmfs index among preschool children in Beijing

Variables	Negative binomial coefficients (95 % CI)	Wald χ2	*P*
Intercept	−0.67 (−1.79, 0.46)	1.35	0.24
Use of nursing bottle at present			
Yes	−0.06 (−0.33, 0.21)	0.20	0.65
No	-----	-----	-----
Frequency of snack consumption			
Never or seldom	−0.23 (−0.90, 0.43)	0.47	0.50
Sometimes (<1 Times/Day)	−0.13 (−0.67,0.41)	0.22	0.63
1–2 Times/Day	−0.24 (−0.76,0.28)	0.82	0.37
3–4 Times/Day	−0.09 (−0.63,0.45)	0.11	0.74
≥5 Times/Day	-----	-----	-----
Frequency of tooth brushing			
Twice daily	0.10 (−0.90, 1.11)	0.04	0.84
Once daily	0.11 (−0.90, 1.11)	0.04	0.84
Less than once daily	−0.07 (−1.11,0.98)	0.02	0.90
Never	-----	-----	-----
Dental visit history			
Yes	0.73 (0.49, 0.97)	34.13	<0.001*
No	-----	-----	-----
MS score in plaque			
0 (CFU <10^4^)	−0.77 (−1.13, −0.42)	18.07	<0.001*
1 (CFU 10^4^–10^5^)	−0.16 (−0.71, 0.38)	0.35	0.55
2 (CFU 10^5^–10^6^)	−0.31 (−0.70, 0.09)	2.29	0.13
3 (CFU >10^6^)	-----	-----	-----
MS score in saliva			
0 (CFU <10^4^)	−0.22 (−0.63, 0.19)	1.11	0.29
1 (CFU 10^4^–10^5^)	−0.19 (−0.60, 0.21)	0.90	0.34
2 (CFU 10^5^–10^6^)	−0.03 (−0.39, 0.33)	0.03	0.87
3 (CFU >10^6^)	-----	-----	-----

*MS* mutans streptococci

*Significant at *P* <0.001

Children with a score of “3” for plaque mutans streptococci (i.e., high level of infection) had 1.97 greater odds of having caries than those who scored “0” (i.e., had low level of infection). Children who had history of dental visit had 1.93 greater odds of having dental caries than children who had not visited a dentist.

Among CF children, a large proportion (60.6 %) had low levels of plaque mutans streptococci (i.e., score = 0), whereas, among ECC children a large proportion (66.3 %) had high level of plaque infection (i.e., score = 3). The prevalence of caries and the mean dmfs score were higher in children with more plaque mutans streptococci (Table 3).

**Table 3:**
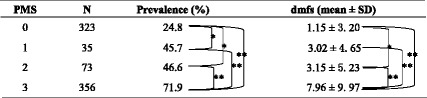
Prevalence of caries and mean dmfs scores for children with different levels of plaque mutans streptococci

*dmfs* decayed, missing or filled surfaces, *PMS* plaque mutans streptococci

* Significant at *P* <0.05; **Significant at *P* <0.001

## Discussion

To our knowledge, this is the first study on the risk factors of ECC in China that has measured mutans streptococci levels in both non stimulated saliva and plaque [2, 18–20]. It is also the first study in China to use the Dentocult SM Strip, a practical and readily used semi-quantitative method, to measure plaque mutans streptococci [21], though a few studies have used this method in other countries [22, 23].

Previous studies have shown that mutans streptococci are strongly associated with dental caries [11, 24]. Vachirarojpisan, et al. [25] found that mutans streptococci level in non stimulated saliva was the only statistically significant indicator of ECC among various variables, which included family income, breast feeding, education level and decayed teeth of caregivers in children aged 6–19 months. Research has also shown that children with caries have higher levels of *Streptococcus mutans* and *Streptococcus sobrinus* in children’ dental plaque [26]. Researchers using PCR have reported a negative association between *Streptococcus mutans* and caries, and instead found *Lactobacillus fermentum* to be associated with ECC [27], indicating that caries is a progressive disease that may be associated with many different caries-causing microorganisms in the course of its development [19].

Many previous studies that reported a positive association between caries and mutans streptococci infection used saliva samples to assess bacterial levels because of the clinical convenience [24, 25]. Some studies also reported that high levels of mutans streptococci and lactobacillus in saliva act synergistically to increase the dmfs index [28]. In our study, both saliva and plaque samples were used to estimate mutans streptococci infection. The results showed that plaque mutans streptococci levels had much stronger correlation with ECC than that in saliva. The positive association that we found between ECC and salivary mutans streptococci level in univariate analysis disappeared on multivariate analysis. Theoretically, plaque is more appropriate for estimating mutans streptococci infection in individuals because tooth surfaces are the natural habitat of this organism [29]. Furthermore, it has been demonstrated that in children aged 9–36 months, the values for mutans streptococci in the dental biofilm are significantly higher than those found in tongue samples [30]. The results of this study are consistent with these previous studies, showing that plaque mutans streptococci levels are superior to salivary levels for indicating risk of ECC among children in Beijing [8].

More than 70 % of the ECC children in this study had dental visit history. Other studies also found higher caries prevalence in children who had visited a dentist [1, 31]. The results of this study suggest that most children visit a dentist only after caries were developed. Parents need to seek preventive care for their children and not just consult the dentist after problems arise.

This study has a few limitations. The WHO diagnostic criteria haven’t included the white spot, and no radiograph was used for diagnosis of proximal caries, thus may underestimate the associations between caries and risk factors. Jin et al. recommended that both noncavitated and cavitated carious lesions should be included in the diagnostic criteria for research of dental caries in primary teeth [32].

The convenience sample selected from an urban area may not represent the overall condition in Beijing. We compared the prevalence of caries in this study (49.0 %) and that of a large-scale survey (46.6 %) containing 226165 3-4-year-old children of Beijing [33], and there was no significant difference. The results of this study are applicable only to other population with similar social background and demographics. Some possible risk factors such as maternal oral health status and enamel hypoplasia were not assessed. In addition, the cross-sectional nature of this study meant that we could only identify correlation between risk factors and caries. A longitudinal study will be necessary to identify and better understand the predictors of ECC.

## Conclusion

In conclusion, this study demonstrates that a high level of mutans streptococci in plaque appears to be a risk factor for ECC in Beijing, China. The plaque level of mutans streptococci is more representative of oral infection and more closely associated with ECC than the non stimulated salivary levels. A longitudinal study is needed to identify any causal relationship between plaque mutans streptococci and ECC in children in Beijing, for it is crucial to screen preschool children at high risk of developing caries.

## Abbreviations

CF: Caries-free
dmfs: Decayed, missing, and filled surfaces
ECC: Early childhood caries
